# BioDWH2: an automated graph-based data warehouse and mapping tool

**DOI:** 10.1515/jib-2020-0033

**Published:** 2021-02-22

**Authors:** Marcel Friedrichs

**Affiliations:** Bielefeld University, Faculty of Technology, Bioinformatics / Medical Informatics Department, Bielefeld, Germany

**Keywords:** data warehousing, database, graph database, pipeline, software tools

## Abstract

Data integration plays a vital role in scientific research. In biomedical research, the OMICS fields have shown the need for larger datasets, like proteomics, pharmacogenomics, and newer fields like foodomics. As research projects require multiple data sources, mapping between these sources becomes necessary. Utilized workflow systems and integration tools therefore need to process large amounts of heterogeneous data formats, check for data source updates, and find suitable mapping methods to cross-reference entities from different databases. This article presents BioDWH2, an open-source, graph-based data warehouse and mapping tool, capable of helping researchers with these issues. A workspace centered approach allows project-specific data source selections and Neo4j or GraphQL server tools enable quick access to the database for analysis. The BioDWH2 tools are available to the scientific community at https://github.com/BioDWH2.

## 1 Introduction

Most studies in life science research require data to conduct different kinds of analyses. With the inception of the OMICS fields such as genomics, transcriptomics, and proteomics each required new, large databases to capture their unique requirements. As the number and complexity of OMICS fields increases such as pharmacogenomics, allergenomics, or Foodomics, the need for more databases and information increases as well. Imker conducted a survey of published databases in the Nucleic Acids Research (NAR) database issues and concluded that as of 2018, 1700 databases were covered in 25 years [1]. The 27th NAR issue from 2019 added 65 new resources and with updates and removals now count 1637 databases [2]. This number only represents the NAR published databases and the total number of databases available online is likely much higher. For the use-case of medical information systems, multiple OMICS levels are relevant in drug therapy safety [3], [4]. Before, it was feasible to only make use of one major pharmacological database, such as “ABDAMED” for the German healthcare market [5]. The growing opportunities of molecular information in the clinical context [6], [7] necessitates the integration of more data sources of high quality from other OMICS fields. This includes finding meaningful relationships between drugs, diseases, and their molecular bases such as gene and protein variants, RNA regulation, and drug pathways. Examples would be the “PharmGKB” [8], “DrugBank” [9], and “OMIM” [10] databases. The integration and mapping of this information could provide an in-depth understanding of individual patient cases and reduce adverse drug reactions towards personalized medicine.

This growth in OMICS fields and data sources necessitates research projects to have a reliable and easy to use integration pipeline for data warehousing and information mapping. Additionally, data sources are heterogeneous in format and availability and have a specific focus to which the format is tailored to. A uniform integration process into a singular data format is therefore beneficial. Another issue is the loose coupling of data sources. Identification systems and external references for entities are included in data sources but can’t guarantee that the referenced data sources will not change. Strong mapping of entities in a data warehouse by introducing a mapping layer and connecting entities from different data sources is another important step. Bringing large amounts of data together helps researchers focus on the analyses they want to perform in one place.

This article presents BioDWH2, an open-source, graph-based data warehouse and mapping tool, helping researchers with data integration and mapping tasks. The goal is a simple setup and execution, and with as little custom configuration as possible.

## 2 Related work

Data integration efforts can be twofold: either a complete integration platform is available for diverse research projects and requirements, or a specific integration workflow is implemented just for one project without being easily reused for other projects. Both have advantages and disadvantages. Project-specific workflows can be fast and focused on requirements. In contrast, complete platforms need to handle a multitude of potential needs. On the other hand, a complete platform may require less knowledge about the integration process from the end-user who in turn can focus on the analyses needed.

Töpel et al. developed the original BioDWH tool for the integration of heterogeneous data sources into a data warehouse [11]. The data sources are integrated into a relational database based on SQL and a simple user interface can guide the user through setup and configuration. No mapping of information is provided as only a simple data warehouse is generated. One important feature is the monitoring of data source updates and consequently updated data integration. With the increasing and widespread use of graph databases [12], [13], [14], using a relational database for large data warehouses may not be best suited anymore. Especially for an additional mapping layer, the analysis of relational tables with joining queries will become very slow and sometimes unfeasible [15]. While graph databases can outperform relational databases, they also provide the opportunity to reveal novel relationships in heterogeneous data [12].

ONDEX/KnetBuilder is part of the larger KnetMiner ecosystem of data integration and analysis tools which has grown significantly over the years [16]. Starting with gene regulatory networks, there are now multiple public instances such as for plants and human diseases, and as an open-source tool can be used by everybody. KnetMiner heavily relies on the Resource Description Framework (RDF) format for graph representation. As RDF is very detailed in their descriptors it can sometimes be more cumbersome to set up and Uniform Resource Identifiers (URI’s) for entities and properties may not be available and have to be created. On the other hand, the mapping between entities is simplified by the uniqueness of entity URI’s. Configuration of the KnetBuilder tool can be more involved, as data source parsers need special Extensible Markup Language (XML) configurations that describe attributes and format-specific properties. Additionally, a workflow has to be created and configured for which files to be used and more.

In contrast to BioDWH and KnetMiner, GenCoNet is a project-specific integration workflow for the analysis of the molecular basis of comorbid diseases hypertension and asthma [17]. Specific data sources of high quality were selected, filtered, and only relevant information integrated into a knowledge graph. The graph is hereby kept small and creating analysis queries is easier, but the extension of the graph with new information as the project continues is slower and requires more effort. Additionally, the database was constructed directly in a Neo4j graph database (https://neo4j.com). While being easy, the workflow depends on the version and continuation of the database system. While Neo4j is unlikely to go away soon, dependence on third party system is always important to be aware of.

## 3 Implementation

BioDWH2 is implemented as a modular open-source Java program, that is easily extensible with new data source modules. An existing installation of the Java Runtime Environment (JRE) 8 is required to run BioDWH2.

### 3.1 BioDWH2 workspace

The main concept for BioDWH2 is the workspace. Because different projects may require different data sources, the workspace allows users to create physically separate data warehouse projects without interfering with previous projects. Additionally, the workspace concept is built with a strict folder structure as visualized in Listing 1. The root folder of the workspace contains a configuration file and a folder “sources” for the data. Each data source resides in a separate folder structure containing the metadata file and a source folder for all the raw data files. Graph files for each data source are generated in the respective data source folder and the final merged and mapped graph files are generated in the “sources” folder.

**Listing 1**

Example of the workspace structure with data sources “PharmGKB” and “HGNC”.

**Figure:**
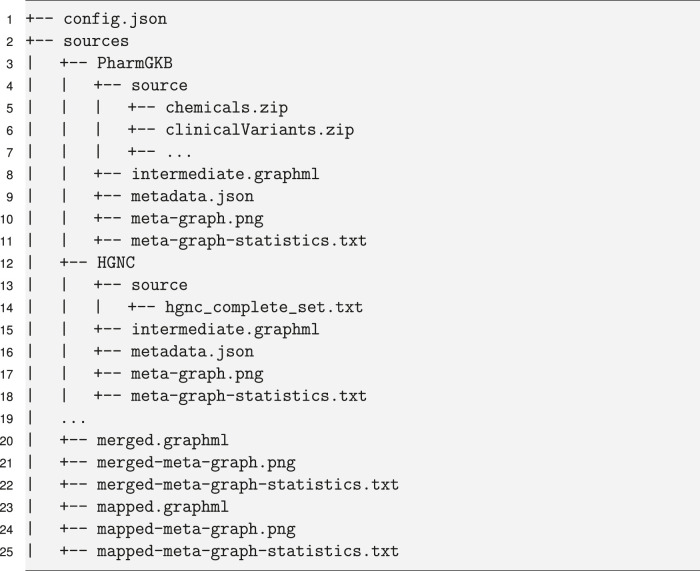

The workspace is configured via a configuration file in JSON format as seen in Listing 2. Version and creation date-time are properties generated by the tool on workspace creation. A version number for the workspace is necessary, to ensure a workspace can be upgraded to newer versions on breaking changes in the future. For end-users, the data source ids property is currently the most relevant one. This field defines which data sources will be integrated into this workspace project by their respective ID. In the example of Listing 2 the data sources “HGNC” [18] and “NDF-RT” [19] are used. Some data sources need additional information to function properly. As an example, the “DrugBank” updater needs the user’s credentials to download the database automatically which can be provided using the data source properties in the configuration file.

**Listing 2**

Example of the JSON encoded configuration file for a workspace.

**Figure:**
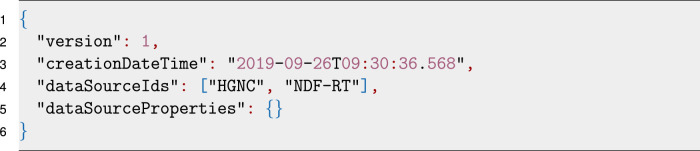

Each data source in the workspace is generated with a metadata JSON file storing relevant status information. An example for the “PharmGKB” data source is visualized in Listing 3. It contains the current version, update timestamp, the source file names downloaded, and flags for each step of the data source processing indicating whether the step was successful.

**Listing 3**

Example of the JSON encoded metadata file for the “PharmGKB” data source.

**Figure:**
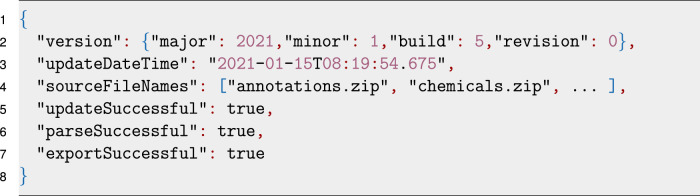

### 3.2 Architecture

**Figure 1:**
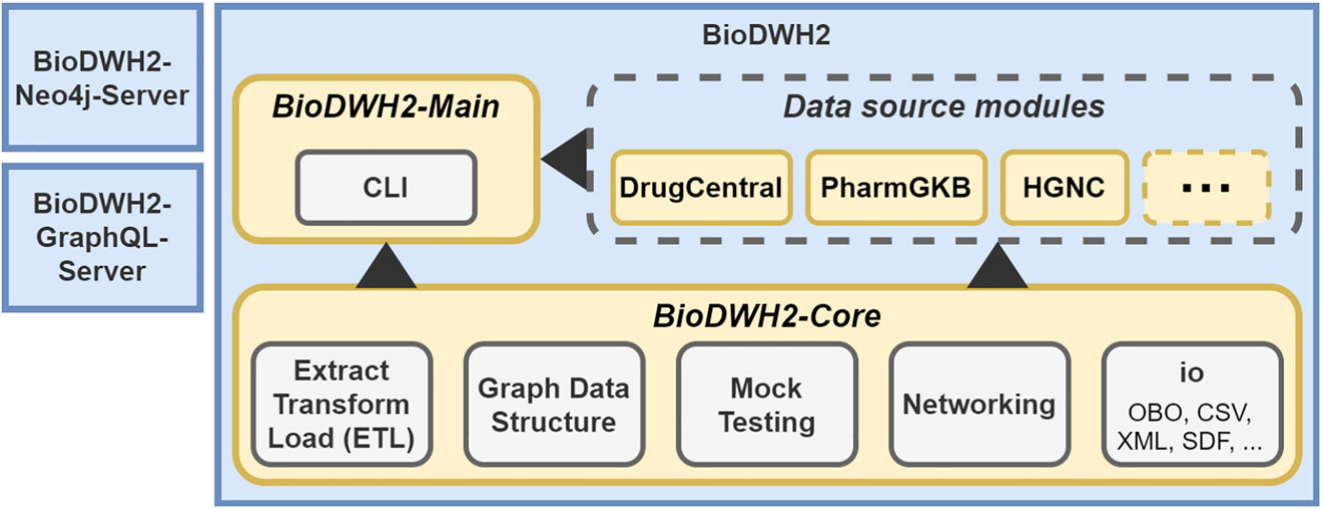
Architecture of BioDWH2 comprised of the core, data source, and main modules. BioDWH2-Neo4j-Server and BioDWH2-GraphQL-Server are tools for using BioDWH2 databases with existing platforms such as Neo4j.

BioDWH2 is designed with a modular architecture to be easily extensible and maintainable. An overview of the architecture is visualized in Figure 1. The central component of the architecture is the BioDWH2-Core, used as a dependency in all other components. BioDWH2-Main is the component referencing the core and all data source modules and provides a simple command-line interface (CLI) for creating and maintaining workspaces. The third component is the data source modules, representing the modular part of the architecture. Each data source is implemented as a separate module, either bundled into the main program during compilation or loaded from a jar file via the java classpath. The end-users interact with the BioDWH2 program via CLI and therefore indirectly with the BioDWH2-Main component.

The BioDWH2-Core provides base classes and utility methods for the implementation of data source modules. The development of new modules is therefore as easy as extending core classes and individually implementing multiple methods for updating, parsing, exporting, and mapping. A set of IO utilities in the core further simplify the implementation process, such as file format parsers for Open Biological and Biomedical Ontology (OBO), XML, structure-data file (SDF), and more. The steps in which data sources are processed are further outlined in the next section.

### 3.3 Program flow

**Figure 2:**
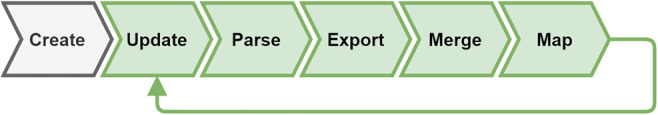
The program flow of BioDWH2 from the creation of a workspace to the final merged and mapped graph database. When new data is available, the process starts over at the update step.

BioDWH2 has a defined program flow used for all workspace projects as visualized in Figure 2. For simplicity the jar file in the following command line listings is called “BioDWH2.jar”. Available downloads of the jar are versioned such as “BioDWH2-v0.1.7.jar”. First, a workspace has to be created at a provided location on the user’s machine using the following command.

**Figure:**
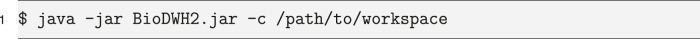

**Figure 3:**
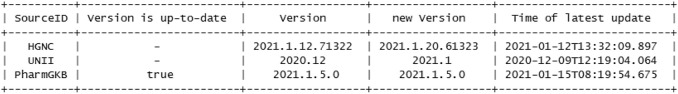
Example output of the BioDWH2 status command.

After the required data sources have been configured in the workspaces “config.json”, either the workspace can be updated, or the status can be checked. Checking the status of the workspace provides detailed information on the configured data sources, whether they are up-to-date, the workspace version, the newest version, and the time of the last update. The status can be checked with the following command and the output is visualized in Figure 3.

**Figure:**
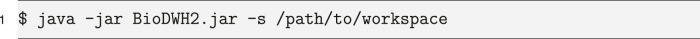

Updating a workspace is split into multiple tasks, of which three are executed sequentially per data source. Starting the update process is done using the following command:

**Figure:**
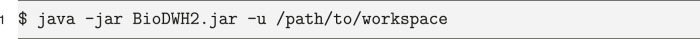

**Figure 4:**
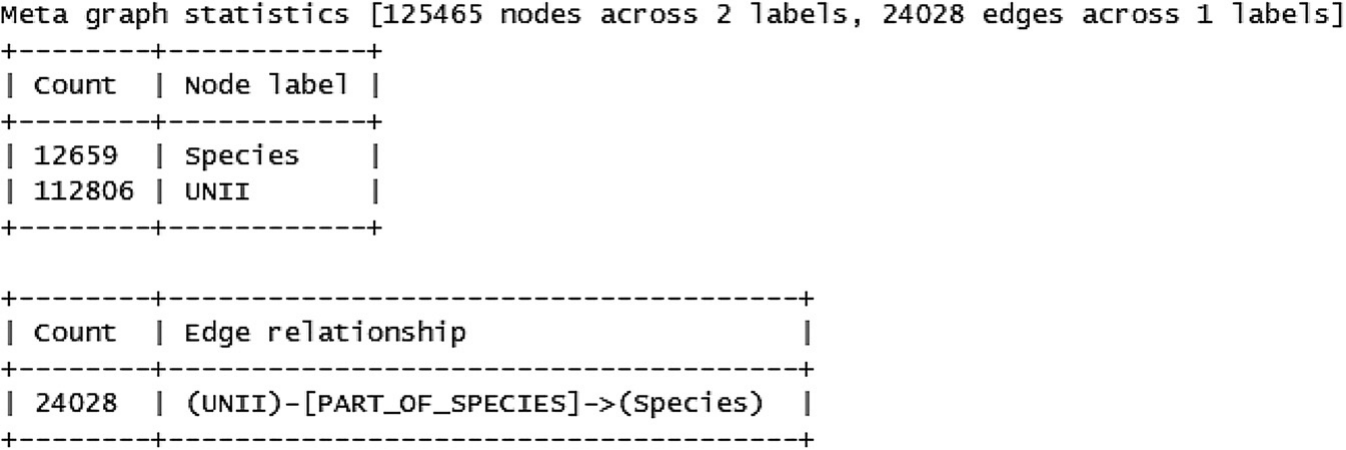
Example meta graph statistics for the “UNII” data source.

First, the data source checks whether a new version is available online and downloads it accordingly to the data source’s “sources” directory. Next, a parser loads all relevant data from the sources which are then used by the exporter. The exporter transforms the raw source data into an internal graph data structure of nodes and edges. Nodes hereby represent entities such as “Drug” or “Gene” and edges their relationships such as “targets” or “is associated with”. The graphs are then stored as intermediates in the data sources directory in Graph markup language (GraphML) format [20]. GraphML was chosen for its simple structure and widespread adoption and interoperability. Additionally, a meta graph for each data source graph is generated and the statistics stored in the data sources directory as “meta-graph-statistics.txt”. An example for the “UNII” [21] data source statistics is visualized in Figure 4. This statistics may help as a first overview of the generated graph. Once all data source modules finished their tasks BioDWH2 collects all intermediate graphs. These are then merged into one large graph which is again stored on disk in GraphML format in the workspace directory. This merged graph already represents a first graph-based data warehouse of all the data sources. As for the data sources, meta graph statistics are generated for the merged graph. Finally, the mapper uses the merged graph and adds a meta-layer of nodes and edges connecting the various data sources where possible. The mapping process is further described in the following section.

### 3.4 Data source mapping

**Figure 5:**
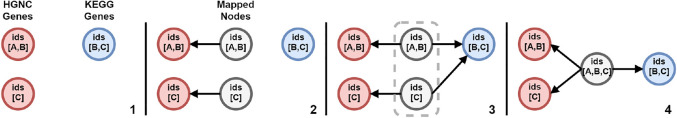
Steps of the node mapping process. (1) The data sources are merged into a single graph. (2) Mapping nodes are created for the first data source and connected with their respective data source nodes. (3) The next data source is mapped resulting in an identifier overlap between two mapping nodes. (4) The overlapping mapping nodes are merged into a singular node.

Mapping data sources in a workspace is split into two parts. First, nodes are mapped by shared identifiers and secondly, edge paths between nodes in each data source are mapped. The core mapping process works without any knowledge of the individual data sources. Instead, data source specific implementations of the “MappingDescriber” class tell the mapper which node labels and edge paths they can describe for their data source. The mapper then uses these descriptions to add nodes and edges in a meta-layer. If multiple entities from different data sources mapped to the same meta-node, these data sources are now interconnected. Nodes that can be described are mapped as visualized in Figure 5. First, a new mapping node is created using the identifiers and label as described by the “MappingDescriber” for a specific node. Then, other mapping nodes with overlapping identifiers are collected and collapsed into a singular mapping node.

**Figure 6:**
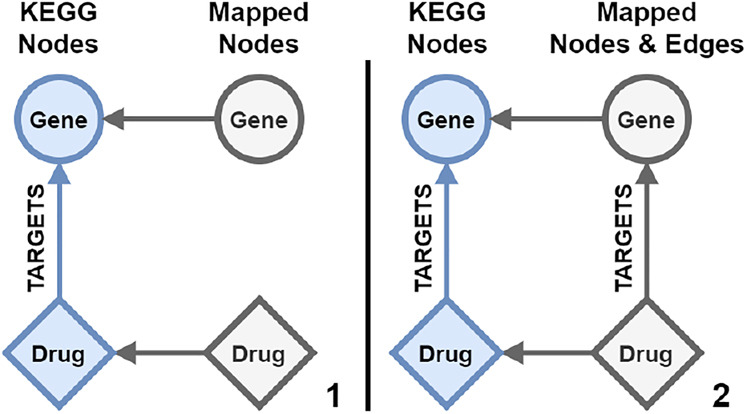
Trivial edge mapping between two mapped data source nodes. (1) Two nodes gene and drug in blue from the same data source are connected with a TARGETS edge. They both are connected to their respective mapping node in grey. (2) A new edge with the mapped label from the TARGETS edge is created between the mapping nodes.

In the trivial case of length one, mapping edge paths would be handled the same as nodes. As a requirement, the two nodes connected by an edge path need to be connected to mapping nodes. The edge can then be mapped as a mapping edge between the two mapping nodes as visualized in Figure 6.

**Figure 7:**
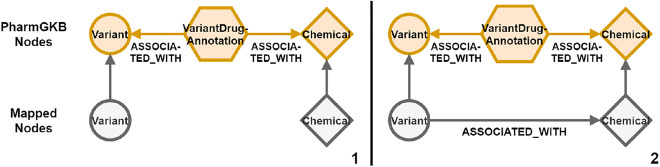
Path mapping of three data source nodes and two edges. (1) Two nodes variant and chemical in orange from the same data source are both associated with a VariantDrugAnnotation node. They both are connected to their respective mapping node in grey. The path of length two is matched and provided to the path mapping. (2) A new edge with the mapped label from the ASSOCIATED_WITH edges is created between the mapping nodes.

However, meaningful relationships between nodes may involve not only a singular edge but a path of edges due to helper or annotation nodes needed for representing the data sources. Mapping singular edges can therefore be seen as an edge path of length one. There is no hard limit on the path length, but longer paths will in turn be more time consuming due to the number of requests on the graph database. The BioDWH2 mapper is handling this requirement by asking the data source’s “MappingDescriber” for paths that should be mapped, as the data source modules should know which paths are important. Paths are hereby represented by a list of labels starting with a node label and alternating between edge and node labels until finishing again with a node label. An example for such a path is the NDF-RT “induces” relationship represented as [“Drug”, “INDUCES”, “Disease”]. These paths are then searched for iteratively by the mapper in the graph. In this example starting from all nodes with the “Drug” label, adjacent edges are searched for which have the label “INDUCES” and are connected to nodes with the “Disease” label. For longer paths this process continues until the full paths are found and the “MappingDescriber” is called to describe each of them. Finally, using this description a new edge is created between the mapping nodes of the first and last node in the path, annotated with the data source id from which it has been mapped. A path example of length two is visualized in Figure 7. The type of mapped paths is up to the data source module developers and should be chosen with great care to not alter the relationships meaning. A set of global relationship names are defined in the BioDWH2 core such as “INDICATES”, “CONTRAINDICATES”, or “INDUCES”. However, any name can be provided to not hinder the development of new data source modules with novel relationship types. The final mapped graph is stored in GraphML format in the workspace directory together with the meta graph statistics.

In this first version of BioDWH2, the user has no direct control over the mapping process, which is planned as a future development. In contrast to combining entities from different data sources, the generated mapping layer is non-destructive of the original data sources. Therefore, the meaning of the data sources is preserved. Another advantage could be the use of the mapping layer separate from the original data sources, for example in an autocomplete field. The mapping layer provides the user with the ability of finding novel relationships between previously disconnected entities and data sources. Finally, if multiple data sources provide relationships between two entities the mapping layer represents them as multiple edges between the respective mapping nodes. This may be used in finding significant relationships by consensus of different data sources in the mapping layer. This does not replace an in-depth analysis of the relationships context and parameters, but may provide a starting point for further analysis.

### 3.5 Data source implementations

Multiple data sources haven already been implemented. These include “HGNC” for genes, “UNII”, “PharmGKB” and “NDF-RT” for pharmacological information, as well as “USDA-PLANTS” [22] for plant species. More data source modules are in development such as the pharmacological databases “DrugCentral” [23] and “DrugBank”. While these data sources are bundled with the BioDWH2 tool and new ones will be added constantly, the development of new data source modules is also available to the end-users. An implementation guide is provided in the GitHub repositories documentation. Community implementations provided as GitHub pull requests are welcome.

### 3.6 Database access

**Figure 8:**
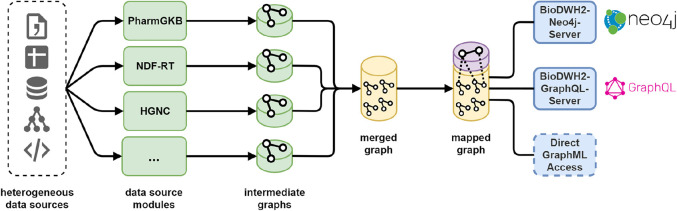
The complete overview of the BioDWH2 data flow from their heterogeneous sources, via the data source modules and intermediate graphs, towards the merged and mapped graphs, and finally the access for analysis.

The main BioDWH2 tool provides integration and mapping, but no analysis capabilities. While analyses may be performed on the mapped GraphML file directly, this may not be feasible for large databases. Therefore, two additional tools are available for accessing the workspace data. The BioDWH2-Neo4j-Server allows for the creation of a Neo4j graph database from the workspace database and running a Neo4j server and browser which are embedded in the tool itself. No setup of a Neo4j server is needed and queries can be run using the Cypher query language directly in the user’s web browser. This allows for a frictionless usage of BioDWH2 for users already familiar with the Neo4j ecosystem. An equivalent BioDWH2-GraphQL-Server is currently in development, to provide a GraphQL (https://graphql.org) endpoint for analysis queries, which directly operate on the workspace database. A complete overview of the data flow is visualized in Figure 8 with access to the data using the aforementioned tools.

## 4 Conclusion

The integration and mapping of heterogeneous data sources is an important first step affecting all subsequent analyses for studies in scientific disciplines. The development of the BioDWH2 tool is intended to ease and simplify this process for researchers and to be usable with limited to no programming skills. The hope is that future research projects can focus more quickly on the analysis instead of integration problems using only the specified data sources needed. As BioDWH2 provides distinct steps in the workflow, users have the option to use it in different ways suitable for their needs. Using the final mapping layer of the data warehouse may provide an easy starting point for analyses. For other users, the mapping layer may not be fitting. They still have the option to use the merged data warehouse and develop their own mapping if needed at all. As a third option users can utilize the BioDWH2 tool to transform data sources into a uniform graph file format. This way they will not have to process the heterogeneous data formats themselves. These options provide the opportunity for a broader user-base to incorporate BioDWH2 into their research projects.

The initial version of BioDWH2 provides robust graph data warehouse and mapping capabilities. However, multiple future developments are planned. The basic analysis tools will be developed to further simplify common analysis tasks and guide researches through the database. While simple meta graph statistics have been implemented, in-depth schema definitions, visualizations, and protocols on how specific entities were mapped need to be implemented and extended. Using the open and widely used GraphQL language will further reduce the barrier of analyzing the resulting graph database. Another important future development will be the possibility to configure certain identifier types as “non-destructive” meaning mapping nodes will not be merged with these identifier types. This would prevent coarse identifier types to cluster mappings with important distinctions.
